# A dynamic optimal control model for COVID-19 and cholera co-infection in Yemen

**DOI:** 10.1186/s13662-021-03271-6

**Published:** 2021-02-15

**Authors:** Ibrahim M. Hezam, Abdelaziz Foul, Adel Alrasheedi

**Affiliations:** 1 Statistics and Operations Research Department, College of Sciences, King Saud University, Riyadh, Saudi Arabia; 2 Department of Mathematics, Ibb University, Ibb, Yemen

**Keywords:** Cholera, Co-infection, COVID-19, Optimal control, Yemen

## Abstract

In this work, we propose a new dynamic mathematical model framework governed by a system of differential equations that integrates both COVID-19 and cholera outbreaks. The estimations of the model parameters are based on the outbreaks of COVID-19 and cholera in Yemen from January 1, 2020 to May 30, 2020. Moreover, we present an optimal control model for minimizing both the number of infected people and the cost associated with each control. Four preventive measures are to be taken to control the outbreaks: social distancing, lockdown, the number of tests, and the number of chlorine water tablets (CWTs). Under the current conditions and resources available in Yemen, various policies are simulated to evaluate the optimal policy. The results obtained confirm that the policy of providing resources for the distribution of CWTs, providing sufficient resources for testing with an average social distancing, and quarantining of infected individuals has significant effects on flattening the epidemic curves.

## Introduction

Recently, Yemen has suffered from many disasters and fierce armed conflicts. It is reported by the UN that Yemen is going through one of the worst humanitarian crises all over the world. The political armed conflict has led to a humanitarian crisis in all fields of life, i.e., more than 85% of the population are suffering from the lack of basic essentials such as food, water, electricity, and medicines. The scarcity of drinkable water has resulted in the spread of infectious diseases such as cholera.

In Yemen, cholera has become the largest epidemic in the modern-day world. According to the World Health Organization (WHO), the cumulative total number of suspected infected individuals from January 2018 to May 2020 is (1,371,819) with 1566 deaths [1]. Cholera is a water-borne bacterial disease which can be transmitted to humans either through humans or water. Several works have investigated the epidemic model of cholera infections, such as Tian and Wang in [2], which implemented some epidemic models for cholera through mathematical analysis. Moreover, the endemic global stability was investigated using three techniques: monotonic dynamical systems, geometric approach, and Lyapunov functions, up to July 17, 2020. Another study is that by Dangbé et al. [3] which identified climatic factors and human behavior parameters that minimize the spread of cholera tangibly or intangibly. The equilibria stabilities of ordinary differential equations in the proposed model were investigated. This work was also applied to some localities of Cameroon and Chad. Likewise, Kobe et al. in [4] suggested a game model of cholera that allows individuals to select one of two programs: vaccinations or clean water consumption. Another related study is that of NKDO Opoku, and Afriyie, C. in [5]. They developed a mathematical model of cholera transmission dynamics of cholera and investigated the following two control measures: education campaign and treatment of water bodies. Berhe in [6] provided a theoretical study of the optimally controlled model of cholera dynamics. The model’s parameters are estimated using cholera data taken from the Oromia region, Ethiopia. Sensitivity analyses were then given for the rate of the infected humans and that of the recovery, as they are the most important parameters. In addition, several techniques were proposed to reduce the number of infected persons and the overall cost associated with each control either separately or both by monitoring the treatment and sanitation parameters.

Furthermore, some studies have discussed the cholera epidemic in Yemen. Based on 2017 real data, Nishiura et al. in [7] predicted the peak of cholera epidemic in Yemen. To estimate the final epidemic size, they used logistic and generalized logistic models. In the same manner, Yang and Wang in [8] updated the susceptible-infected-recovered (SIR) model to include a parameter measuring the availability of medical resources and facilities. They also provided a mathematical analysis of the proposed model. The proposed model was implemented on the real data taken from a cholera epidemic in Yemen from April 2017 to May 2018. Enhancing the same study, Lemos-Paião et al. in [9] introduced vaccination to the SIR model as a parameter for optimal control. This model was applied to the cholera outbreak in Yemen from April 27, 2017 to April 15, 2018. Based on the same data taken in the same period, Lemos-Paião et al. in [10] updated the SIR model to require quarantining during the treatment period. Also, one parameter measure was used for optimal control, corresponding to the ratio of susceptible individuals receiving the chlorine water tablets (CWTs) for water purification. The next related study concering cholera outbreak in Yemen in the same period was carried by Carfora and Torcicollo in [11]. In their study, the SIR model considered parameters that reflect direct (human-to-human) and indirect (environment-to-human) spread. The approximation approach of the least squares was also used to estimate the epidemiological parameters.

All the above studies have to do with cholera, on the other side; there are some that appeared with the outbreak of COVID-19. The first confirmed case of COVID-19 in Yemen was discovered in April 2020. Since then, the cumulative total number of infected cases and deaths due to COVID-19 up to June 17, 2020 are 902 and 244 cases, respectively. It should be noted that the announced cases are much less than the actual number of cases in Yemen due to many reasons (e.g., political or technical). Dozens of mathematical models have been proposed to control the dynamics of the COVID-19 pandemic. Madubueze in [12] updated the SEQIHR (susceptible-exposed-quarantined-infected-hospitalized-recovered) model to include new parameters reflecting the impact of health education, quarantine, and isolation. These parameters were used to determine the optimal control and to find the minimum cost associated with each control. As to Khajji et al. in [13], the proposed mathematical model included the dynamic transmission of COVID-19 between humans and animals in a region or in several regions at discrete times. They presented some control strategies to protect the maximum number of individuals. Hence, the least cost and most effective strategy to be determined. Perkins and Espana in [14] presented an optimal control analysis of the susceptible-exposed-asymptomatic-infected-hospitalized-vaccinated (SEAIHV) model of COVID-19 dynamic transmission. They also added a parameter representing non-pharmaceutical interventions. The optimal control priority was to minimize the total number of deaths versus reduced time under control. The validity of the model was tested based on the real data taken from US, from May 2020 through December 2021. Likewise, Wickramaarachchi and Perera in 2020 [15] expanded the susceptible-exposed-infected-recovered (SEIR) model. They classified the infected people into four groups: asymptomatic patients, patients with mild symptoms, hospitalized patients, and critical patients. They also used two parameters to reflect personal protection rate: asymptomatic people identification rate and tracking rate. The proposed model was implemented to fight the COVID-19 outbreak in Sri Lanka. Yousefpour et al. [16] proposed the susceptible-exposed-asymptomatic-infected-hospitalized-recovered (SEAIHR) model. They designed a multi-objective genetic algorithm for optimal control of economic consequence strategies. Tsay et al. in [17] expanded the susceptible-exposed-asymptomatic-infected-removed (SEAIHR) model to include the perished class due to COVID-19 infection. Besides, an analytical comparison was presented to estimate the system parameters for USA, Italy, Spain, and Germany. Social distancing, extensive testing, and quarantining were used for dynamic optimal control. The model aimed at minimizing social and economic costs so as to maintain the size of the epidemic below its specific peak value. Also, the authors in [18–21] presented interesting mathematical models that discussed controlling the spread of COVID-19 and allocation of COVID-19 vaccines to priority groups using the MCDM approach. Besides, several studies have provided mathematical analyses of other infectious diseases such as TB [22–24], Ebola [25], HBV [26], HIV [27], Lassa hemorrhagic fever [28], dengue [29], etc.

Recently, some studies on co-infection models have been carried out. Co-infection is simultaneous infection of an individual due to the outbreak of more than one infectious disease in the same place. The first related study to be indicated here is that of Li et al. [30]. They proposed an epidemiological model concerned with co-infection with two diseases where one of them is chronic, the other is acute. Similarly, Gao et al. [31] developed and analyzed a simple susceptible-infected-susceptible (SIS) model of co-infections. Similarly, Tang et al. [32] proposed a mathematical model of dual-infection of dengue and Zika virus. Again, Ghersheen et al. [33, 34] proposed SIR models to describe dual infection in the same area. Also, Khan et al. [35] proposed a co-infection model that includes the Atangana–Baleanu fractional derivative, which is a combination of the human immunodeficiency virus (HIV) and tuberculosis (TB). Besides, Mushayabasa and Bhunu [36] proposed a mathematical model concerning the relationship between HIV and cholera outbreaks. Further, Okosun and Makinde in [37] formulated an SIR model for the concurrent infection of malaria and cholera. The authors discussed five parameters for optimal control: two of them were for preventing both diseases, the other two were for controlling the treatment of each disease, and the last one for controlling the co-infection treatment. Equally important, Okosun et al. [38] proposed a compartmental model to address the co-infection dynamics of cholera and schistosomiasis diseases. In the same way, Marimuthu et al. [39] investigated the impact of COVID-19 on TB patients using the SEIR model in India. According to public health interventions, two policies were studied to estimate the basic reproduction number. Lam et al. [40] wrote a letter for controlling the simultaneous outbreak of dengue and COVID-19 in Singapore. Doungmo et al. in [41] discussed the coinfection of HIV with the COVID-19 based on a mathematical model, and Hezam in [42] combined the COVID-19 model and the unemployment problem, while Zhang and Jain in [43] investigated the transmission of the Ebola and the Covid-19 viruses.

However, studies on mathematical models of cholera infection with other infectious diseases are rare. At the time of writing this paper, mathematical model studies of COVID-19 infection with other diseases are almost scarce. Besides, there is no epidemiological model in the literature so far that implements the co-infection dynamics of cholera and COVID-19. On the other hand, the simultaneous outbreak of both diseases in Yemen overwhelms the fragile health care system. In fact, the control variables used to encounter any infection contribute indirectly to fighting another infection. Filling swamps, purifying water along with pure environment and personal hygiene, for example, assist to curb cholera outbreak, on one hand, and on the other they are significant factors in fighting COVID-19. Likewise, social distancing leads to curbing COVID-19 and helps also to control cholera outbreak. Therefore, we need to propose an optimal control model that combines two simultaneous epidemics in the same region. Hence, this is the founding motivation of this study.

In this study, we present a formulation consisting of dynamic ordinary differential equations of an epidemiological co-infection model. This work provides the following significant contributions. Firstly, we propose a framework for mathematical model that integrates COVID-19 and cholera diseases. Secondly, based on the actual data about both infections from January 1, 2020 to May 30, 2020, we estimate parameters so as to predict the trajectories of both outbreaks for 100 weeks. Thirdly, we propose an optimal control model to minimize both the expected cumulative number of people infected with COVID-19 and cholera, and the total cost associated with each control. Fourthly, by testing a novel set of policies, we examine the responsiveness of the optimum of control inputs. We give special emphasis on inputs related to social distancing, lockdown, number of tests, and chlorine availability in order to determine the optimal policies that lead to infection mitigation. Fifthly, all the policies that have to do with COVID-19 and cholera outbreaks in Yemen are carried out. Ultimately, under the current conditions and available resources in Yemen, we determine the optimal policy.

What follows in this work is organized as follows. Section 2 discusses the co-infection model formulation. Section 3 discusses the estimated parameters problem. In Sect. 4, the optimal control model and its analysis are provided. In Sect. 5, numerical simulations are presented. Finally, in Sect. 6, the work is summarized and concluded.

## Model formulation

This study builds upon the models presented in [10, 11, 17]. Tsay et al. in [17] discussed dynamic models of COVID-19, and other studies addressed cholera outbreak in Yemen. To investigate the co-infection dynamics of COVID-19 and cholera, we subdivide the total human population into fourteen different epidemiological classes whose descriptions are found in Table 1. Furthermore, Table 2 summarizes the parameters used in the co-infection model.

**Table 1:** Descriptions and initial values of model variables

Variables	Description	Initial conditions	Source
*N*	Total population size	29,825,964	[44]
*S*	Susceptible to both COVID-19 and cholera	*N*	Assumed
*E*	Number of exposed to COVID-19	[0,*N* × 10^−6^]	[17]
*A*	Number of asymptomatic COVID-19 individuals	0	Assumed
$I_{1}$	Number of COVID-19- infected individuals	0	Assumed
$R_{1}$	Recovered from COVID-19	0	Assumed
$P_{1}$	Perished by COVID-19	0	Assumed
$I_{R2}$	Infected with cholera after recovery from COVID-19	0	Assumed
$I_{2}$	Infected with cholera	$I_{0} = 750$ person	[10]
$R_{2}$	Recovered from cholera	0	Assumed
$P_{2}$	Perished by cholera	0	Assumed
$I_{1R}$	Infected with COVID-19 after recovery from cholera	0	Assumed
$I_{12}$	Co-infected with both cholera and COVID-19	0	Assumed
$R_{12}$	Recovered from both cholera and COVID-19	0	Assumed
$P_{12}$	Perished by both COVID-19 and cholera	0	Assumed
*B*	Bacterial concentration in the environment (free bacteria population living in the environment)	275 × 10^3^ (cell/ml)	[10]

**Table 2:** Descriptions and values of model parameters

Parameters	Description	Value (range)	Source
$I_{1}^{\mathrm{peak}}$	Peak limit of COVID-19	[10^4^,10^5^]	Assumed
$I_{2}^{\mathrm{peak}}$	Peak limit of cholera	[10^5^,5 × 10^5^]	Assumed
$u_{1} ( t )$	Time-dynamic function to measure the social distancing rate	[0.05,0.5]	[17]
$u_{2} ( t )$	Time-dynamic function to measure the quarantining rate	[0.01,0.3]	[17]
$u_{3} ( t )$	Time-dynamic function to measure the testing rate	[0.1,0.3]	[17]
$u_{4} ( t )$	Time-dynamic function to measure the fraction of susceptible individuals who have access to CWT for water purification	[0.2,1]	[10]
$t_{\mathrm{latent}}^{-1}$	Latent period of the virus	0.5 days^−1^	[17]
$\alpha _{c} ( t )$	Direct transmission rate of cholera	$5.5 e^{-7}$	[11]
*β*	Indirect transmission rate of cholera	0.02325/week	Estimated
$K_{B}$	Half saturation constant	10^6^ (cell/ml)	[11]
$\mu _{1}$	Susceptible to COVID-19 after recovering	0,	[17]
$\mu _{2} $	Susceptible to cholera after recovering	0.0003/week,	[11]
$\mu _{12}$	Susceptible to both COVID-19 and cholera after recovering	0	Assumed
*ρ*	Infectious period for subjects with unconfirmed infections	0.1 days^−1^	[17]
$\delta _{1}$	Infection rate with cholera from COVID-19 patient	0	Estimated
$\beta _{1}$, $\beta _{2}$	Recovery rate from COVID-19 and cholera	0.005, 1.5	Estimated
$\lambda _{1}$	Infected rate of cholera after recovery from COVID-19	0	Estimated
$\upsilon _{1}$	Recovery rate from COVID-19 and cholera sequentially	0	Estimated
$\omega _{1}$, $\omega _{2}$, $\omega _{12}$	Death rate by COVID-19, cholera, and both	0.0447, 0.0002, 0	Estimated
$\alpha _{12}$	Infected rate by both cholera and COVID-19 simultaneously	0	Estimated
$\delta _{2}$	Infected rate with COVID-19 from cholera patient.	0	Estimated
*ξ*	Shedding rate (infected)	70 (cell/ml week^−1^ person^−1^)	[11]
*τ*	Bacteria death rate	0.233 /week	[11]
$\lambda _{2}$	Infected rate by COVID-19 after recovering from cholera	0	Estimated
$\upsilon _{2}$	Recovery rate from cholera and COVID-19 sequentially	0	Estimated
$\upsilon _{12}$	Recovery rate from both COVID-19 and cholera simultaneously	0	Estimated
*k*	Bacteria carrying capacity	$K_{B} /2$	[11]
*b*	Allee threshold when *τ* = 0	$K_{B} /10$	[11]
*r*	Bacterial intrinsic growth rate	1	[11]
Λ	A constant rate of the total population	0.3	Assumed

The variables $S ( t )$, $E ( t )$, $A ( t )$, $I_{1} ( t )$, $R_{1} ( t )$, $P_{1} ( t )$, $I_{R2} ( t )$, $I_{2} ( t )$, $R_{2} ( t )$, $P_{2} ( t )$, $I_{1R} ( t )$, $I_{12} ( t )$, $R_{12} ( t )$, and $P_{12} ( t )$ correspond to the numbers of individuals in the fourteen epidemiological classes at time *t*. The total human population at time *t*, denoted by $X ( t )$, is given by

$$ \begin{aligned} X ( t ) &= S ( t ) +E ( t ) +A ( t ) + I_{1} ( t ) + R_{1} ( t ) + P_{1} ( t ) + I_{R2} ( t ) + I_{2} ( t ) + R_{2} ( t ) + P_{2} ( t )\\ &\quad {} + I_{1R} ( t ) + I_{12} ( t ) + R_{12} ( t ) + P_{12} ( t ). \end{aligned} $$

Moreover, the total human population is combined with the pathogen population $B ( t )$ in the environment, where $B ( t )$ reflects the bacterial concentration for cholera infection dynamics at time *t*.

**Figure 1:**
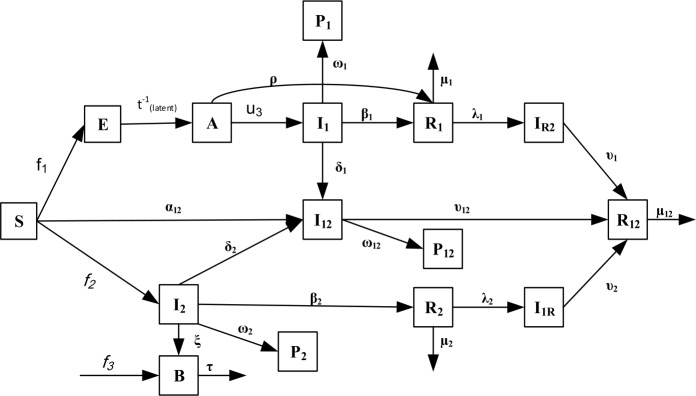
Schematic diagram of the compartmental cholera-COVID-19 co-infection model. Please note that $f_{1} = ( 1- u_{1} ( t ) ) A ( t ) + ( 1- u_{2} ( t ) ) I_{1} ( t )$, $f_{2} = \alpha _{c} ( t ) I_{2} ( t ) + \frac{\beta B}{K_{B} +B} ( 1- u_{4} ( t ) )$ and $f_{3} = rB ( B-b ) ( 1- \frac{B}{k} )$ in the flow chart

The schematic diagram of the compartmental COVID-19-cholera co-infection model is shown in Fig. 1.

The proposed co-infection model is described by the following system of equations:

1 $$\begin{aligned} & \frac{dS ( t )}{dt} =- \biggl( \frac{ ( 1- u_{1} ( t ) )}{N} A ( t ) + \frac{ ( 1 - u_{2} ( t ) )}{N} I_{1} ( t ) + \alpha _{c} ( t ) I_{2} ( t ) \\ &\hphantom{\frac{dS ( t )}{dt} =}{} + \frac{\beta B}{K_{B} +B} \bigl( 1- u_{4} ( t ) \bigr) + \alpha _{12} \biggr) S ( t ) + \mu _{1} R_{1} ( t ) + \mu _{2} R_{2} ( t ) + \mu _{12} R_{12} ( t ) + \Lambda, \end{aligned}$$

2 $$\begin{aligned} & \frac{dE ( t )}{dt} = \biggl( \frac{ ( 1- u_{1} ( t ) )}{N} A ( t ) + \frac{ ( 1- u_{2} ( t ) )}{N} I_{1} ( t ) \biggr) S ( t ) - t_{\mathrm{latent}}^{-1} E ( t ), \end{aligned}$$

3 $$\begin{aligned} & \frac{dA ( t )}{dt} = t_{\mathrm{latent}}^{-1} E ( t ) - \bigl( u_{3} ( t ) +\rho \bigr) A ( t ), \end{aligned}$$

4 $$\begin{aligned} & \frac{d I_{1} ( t )}{dt} = u_{3} ( t ) A ( t ) - ( \delta _{1} + \beta _{1} + \omega _{1} ) I_{1} ( t ), \end{aligned}$$

5 $$\begin{aligned} & \frac{d R_{1} ( t )}{dt} =\rho A ( t ) + \beta _{1} I_{1} ( t ) - ( \mu _{1} + \lambda _{1} ) R_{1} ( t ), \end{aligned}$$

6 $$\begin{aligned} & \frac{d P_{1} ( t )}{dt} = \omega _{1} I_{1} ( t ), \end{aligned}$$

7 $$\begin{aligned} & \frac{d I_{R2} ( t )}{dt} = \lambda _{1} R_{1} ( t ) - \upsilon _{1} I_{R2} ( t ) , \end{aligned}$$

8 $$\begin{aligned} & \frac{d I_{12} ( t )}{dt} = \alpha _{12} S ( t ) + \delta _{1} I_{1} ( t ) + \delta _{2} I_{2} ( t ) - ( \upsilon _{12} + \omega _{12} ) I_{12} ( t ), \end{aligned}$$

9 $$\begin{aligned} & \frac{d R_{12} ( t )}{dt} = \upsilon _{1} I_{R2} ( t ) + \upsilon _{2} I_{1R} ( t ) + \upsilon _{12} I_{12} ( t ) - \mu _{12} R_{12} ( t ), \end{aligned}$$

10 $$\begin{aligned} & \frac{d P_{12} ( t )}{dt} = \omega _{12} I_{12} ( t ), \end{aligned}$$

11 $$\begin{aligned} & \frac{d I_{2} ( t )}{dt} = \biggl( \alpha _{c} ( t ) I_{2} ( t ) + \frac{\beta B}{K_{B} +B} \bigl( 1- u_{4} ( t ) \bigr) \biggr) S ( t ) - ( \delta _{2} + \beta _{2} + \omega _{2} +\xi ) I_{2} ( t ) , \end{aligned}$$

12 $$\begin{aligned} & \frac{dB ( t )}{dt} =rB ( B-b ) \biggl( 1- \frac{B}{k} \biggr) -\tau B+\xi I_{2} ( t ) , \end{aligned}$$

13 $$\begin{aligned} & \frac{d R_{2} ( t )}{dt} = \beta _{2} I_{2} ( t ) - ( \mu _{2} + \lambda _{2} ) R_{2} ( t ) , \end{aligned}$$

14 $$\begin{aligned} & \frac{d P_{2} ( t )}{dt} = \omega _{2} I_{2} ( t ), \end{aligned}$$

15 $$\begin{aligned} & \frac{d I_{1R} ( t )}{dt} = \lambda _{2} R_{2} ( t ) - \upsilon _{2} I_{1R} ( t ). \end{aligned}$$

Equation (1) describes individuals susceptible to both COVID-19 and cholera. Inputs for this epidemiological class are the fraction of the total population at a constant rate of Λ and the fraction of recovered individuals from COVID-19, cholera, and both diseases with rates of $\mu _{1}$, $\mu _{2}$, and $\mu _{12}$, respectively, minus individuals newly exposed to COVID-19 and cholera. The rates of exposure to COVID-19 are governed by the time-dependent inputs $u_{1} ( t )$ and $u_{2} ( t )$, which correspond to the social measures taken during the course of the COVID-19 pandemic. While $u_{1} ( t )$ refers to the rate of social distancing of asymptomatic carriers (*A*), $u_{2} ( t )$ refers to the rate of quarantining of infected people ($I_{1}$). On the other hand, the rate of incidence of cholera is governed by direct and indirect transmission rates. While human-to-human interaction forms the direct transmission $\alpha _{c} ( t )$, the environment-to-human transmission forms the indirect transmission $\frac{\beta B}{K_{B} +B}$. The time-dependent input $u_{4} ( t )$ corresponds to the fraction of susceptible individuals who have access to CWT for water purification.

Equation (2) describes individuals exposed to COVID-19. This epidemiological class includes a fraction of susceptible individuals minus individuals exposed during the latent period. Equation (3) describes individuals infected with COVID-19 but still asymptomatic or unconfirmed. The individuals of this epidemiological class come from the exposed class at a rate of $t_{\mathrm{latent}}^{-1}$. This class will be left either by confirmation of COVID-19 infection or by direct recovery. Equation (4) describes confirmed infected individuals that have been tested. The screening level is measured by a time-dependent parameter $u_{3} ( t )$. Thus, $u_{3} ( t )$ is added to the other control variables $u_{1} ( t )$, $u_{2} ( t )$, $u_{3} ( t )$ that will be used to verify the various measures taken during the COVID-19 epidemic. This epidemiological class will be left either for recovery at a rate of $\beta _{1}$, or for death at a rate of $\omega _{1}$, or for co-infection at a rate of $\delta _{1}$. Equation (5) describes individuals recovered from COVID-19 with or without symptoms. This class is increased due to the recovery of infected people in both classes from COVID-19 at rates of $\beta _{1}$ and *ρ*, respectively. On the other hand, this class is decreased due to infection with cholera or reinfection with COVID-19 at rate of $\lambda _{1}$ or $\mu _{1}$ respectively. Equation (6) describes individuals perished by COVID-19 at a rate of $\omega _{1}$. Equation (7) describes infected individuals with cholera after recovering from COVID-19. This class is increased when the COVID-19 recovered individuals get infected with cholera at a rate of $\lambda _{1}$. It is reduced by recovering from COVID-19 and cholera respectively at a rate of $\upsilon _{1}$. Equation (8) describes individuals infected with both COVID-19 and cholera, either consecutively or simultaneously. The inputs to this class are either the infection of susceptible individuals or because a patient with one infection is infected with the other one at rates of $\alpha _{12}$, $\delta _{1}$, and $\delta _{2}$, respectively. It is decreased by the individuals’ recovery at a rate of $\upsilon _{12}$ or individuals’ death at rate of $\omega _{12} $. Equation (9) describes individuals recovered from both COVID-19 and cholera, either consecutively or simultaneously. It is increased when infected individuals recover from cholera, COVID-19, or both at rates of $\upsilon _{1}$, $\upsilon _{2}$, and $\upsilon _{12}$, respectively. It is decreased by natural death or becoming susceptible to both infections at a rate of $\mu _{12} $. Equation (10) describes individuals perished due to both COVID-19 and cholera at a rate of $\omega _{12}$. Equation (11) describes individuals infected with cholera. Cholera can be transmitted either directly through human’s interaction at a rate of $\alpha _{c} ( t )$ or by indirectly through bacteria ingestion with per capita contact rate of *β*. The probability of transmission from the environment is represented by $\frac{\beta B}{K_{B} +B}$, where $K_{B}$ is the level of pathogen concentration whereabouts half of all contacts with impure water produce infection and is related to bacteria concentration *B* in the water. Therefore, the risk of infection increases as long as *B* increases; and it decreases as $u_{4} ( t )$ increases. People in this class have three options for leaving this class either to get dual infection at a rate of $\delta _{2}$, or to get recovered from cholera at a rate of $\beta _{2}$, or die due to cholera at a rate of $\omega _{2}$.

Equation (12) describes the environmental free bacteria. It is increased by the infected individuals at a rate of *ξ*, where humans can release bacteria into the open environment. This epidemiological class is increased by bacterial growth, which can be described by the logistic equation $rB ( B - b ) ( 1- \frac{B}{k} )$, where *r* is the intrinsic growth, *k* is the capacity of bacteria carrier, and *b* is the Allee threshold when $\tau =0$. This epidemiological class is decreased by the death of bacteria with a mortality rate of *τ*. Equation (13) describes individuals recovered from cholera at a rate of $\beta _{2}$. This epidemiological class is decreased by returning to being susceptible to both diseases or COVID-19 at rates of $\mu _{2} $ and $\lambda _{2}$, respectively. Equation (14) describes individuals dying due to cholera at a rate of $\omega _{2}$. Equation (15) describes individuals infected with COVID-19 after recovery from cholera at a rate of $\lambda _{2}$. The input of this class comes when the cholera recovered individuals are infected with COVID-19 at a rate of $\lambda _{2}$. Also, this class will be left by recovering from cholera and COVID-19 respectively at a rate of $\upsilon _{2}$. It is worth noting that the parameters in this work are classified into three categories. The first category includes time-varying inputs that reflect different rates of social distancing, quarantine, COVID-19 testing kit, and CWT for water purification. The second one includes values to be estimated from the real data obtained from Yemen such as transmission rate, recovery rate, and death rate. The third category includes literature-based values.

Furthermore, we can prove that the system is well defined as the classes’ population are positive and bounded.

### Lemma 1

(Positivity of the solution)

*Let the initial conditions of model system* (1)*–*(15) *be nonnegative*. *Then the solutions of the proposed system are also nonnegative for all* $t>0$

$$\begin{aligned}& \begin{gathered} S ( t ) \geq 0,\qquad E ( t ) \geq 0,\qquad A ( t ) \geq 0,\qquad I_{1} ( t ) \geq 0,\qquad R_{1} ( t ) \geq 0,\\ P_{1} ( t ) \geq 0,\qquad I_{R2} ( t ) \geq 0,\qquad I_{2} ( t ) \geq 0,\qquad R_{2} ( t ) \geq 0,\qquad P_{2} ( t ) \geq 0,\\ I_{1R} ( t ) \geq 0,\qquad I_{12} ( t ) \geq 0,\qquad R_{12} ( t ) \geq 0,\qquad P_{12} ( t ) \geq 0\quad \forall t>0. \end{gathered} \end{aligned}$$

### Proof

Let us take $t_{1}$ as

$$ \begin{aligned} t_{1} &= \sup \bigl\{ t>0: S ( \tau ) >0,E ( \tau ) >0,A ( \tau ) >0, I_{1} ( \tau ) >0, R_{1} ( \tau ) >0, P_{1} ( \tau ) >0, \\ &\quad I_{R2} ( \tau ) >0, I_{2} ( \tau ) >0, R_{2} ( \tau ) >0, P_{2} ( \tau ) >0, I_{1R} ( \tau ) >0, I_{12} ( \tau ) >0,\\ &\quad R_{12} ( \tau ) >0, P_{12} ( \tau ) >0\ \forall \tau \in [ 0,t ] \bigr\} . \end{aligned} $$

Consider $S ( 0 ) \geq 0$, $E ( 0 ) \geq 0$, $A ( 0 ) \geq 0$, $I_{1} ( 0 ) \geq 0$, $R_{1} ( 0 ) \geq 0$, $P_{1} ( 0 ) \geq 0$, $I_{R2} ( 0 ) \geq 0$, $I_{2} ( 0 ) \geq 0$, $R_{2} ( 0 ) \geq 0$, $P_{2} ( 0 ) \geq 0$, $I_{1R} ( 0 ) \geq 0$, $I_{12} ( 0 ) \geq 0$, $R_{12} ( 0 ) \geq 0$, $P_{12} ( 0 ) \geq 0$.

Now, let us take Eq. (1) as an example:

$$ \begin{aligned} \frac{dS ( t )}{dt} &=- \biggl( \frac{ ( 1- u_{1} ( t ) )}{N} A ( t ) + \frac{ ( 1- u_{2} ( t ) )}{N} I_{1} ( t ) + \alpha _{c} ( t ) I_{2} ( t )\\ &\quad {} + \frac{\beta B}{K_{B} +B} \bigl( 1- u_{4} ( t ) \bigr) + \alpha _{12} \biggr) S ( t ) + \mu _{1} R_{1} ( t ) + \mu _{2} R_{2} ( t ) + \mu _{12} R_{12} ( t ) + \Lambda . \end{aligned} $$

For simplicity, we are assuming that

$$ f_{4} ( s ) = \biggl( \frac{ ( 1- u_{1} ( t ) )}{N} A ( t ) + \frac{ ( 1- u_{2} ( t ) )}{N} I_{1} ( t ) + \alpha _{c} ( t ) I_{2} ( t ) + \frac{\beta B}{K_{B} +B} \bigl( 1- u_{4} ( t ) \bigr) + \alpha _{12} \biggr). $$

Thus, $\frac{dS ( t )}{dt} + f_{4} ( s ) S ( t ) = \mu _{1} R_{1} ( t ) + \mu _{2} R_{2} ( t ) + \mu _{12} R_{12} ( t ) + \Lambda $.

This leads to

$$ \frac{d}{dt} \biggl( S ( t ) \exp \biggl( \int _{0}^{t_{1}} f_{4} ( s ) \,ds \biggr) \biggr) = \bigl( \mu _{1} R_{1} ( t ) + \mu _{2} R_{2} ( t ) + \mu _{12} R_{12} ( t ) +\Lambda \bigr) \exp \biggl( \int _{0}^{t_{1}} f_{4} ( s ) \,ds \biggr). $$

Hence, we get on

$$ S ( t ) = \exp \biggl( - \int _{0}^{t_{1}} f_{4} ( s ) \,ds \biggr) \biggl[ \int _{0}^{t_{1}} \bigl( \mu _{1} R_{1} ( t ) + \mu _{2} R_{2} ( t ) + \mu _{12} R_{12} ( t ) +\Lambda \bigr) \exp \biggl( \int _{0}^{t_{1}} f_{4} ( s ) \,ds \biggr) \biggr]. $$

This proves that $S ( t ) >0$ for all $t >0$. In the same manner, we can prove that for all fifteen epidemiological classes. □

### Lemma 2

*The solutions of system* (1)*–*(15) *are bounded*.

In the proposed system (1)–(15), we have two different populations, the human and the bacteria population.

Using the fact that the total human population at time *t* is $X ( t )$ and the reduced system of system (1)–(15) except (12), we obtain:

$$ \frac{dX ( t )}{dt} = \Lambda -\xi I_{2} ( t )\quad \Rightarrow\quad \frac{dX ( t )}{dt} \leq \Lambda . $$

By integrating both sides of the above equation, we have

$$ \Rightarrow X ( t ) \leq \Lambda + X_{0}. $$

Since each human epidemiological class is less than $X ( t )$, all human classes are bounded.

Hence all the solutions of system (1)–(15) related to the human and that initiating in $\{ \mathbb{R}_{+}^{14} \backslash 0 \} $ are confined in the region

$$ \begin{aligned} \Omega &= \bigl\{ \bigl( S ( t ),E ( t ),A ( t ), I_{1} ( t ), R_{1} ( t ), P_{1} ( t ), I_{R2} ( t ), I_{2} ( t ), R_{2} ( t ), P_{2} ( t ),\\ &\quad I_{1R} ( t ), I_{12} ( t ), R_{12} ( t ), P_{12} ( t ) \bigr) \in \mathbb{R}_{+}^{14}:X< \Lambda +\varepsilon , \vert \varepsilon >0, t\rightarrow \infty \bigr\} . \end{aligned} $$

Now, we have some cases for the bacteria class $B ( t )$.

From (12), we have

$$ \frac{dB ( t )}{dt} =rB ( B-b ) \biggl( 1- \frac{B}{k} \biggr) -\tau B+\xi I_{2} ( t ). $$

Since $I_{2} ( t ) \leq \Lambda +\varepsilon $ for all *t*, we obtain

$$ \frac{dB ( t )}{dt} \leq rB ( B-b ) \biggl( 1- \frac{B}{k} \biggr) -\tau B+ \xi ( \Lambda +\varepsilon ). $$

In the absence of an infected case [11], we set

$$ f ( B ) =rB ( B-b ) \biggl( 1- \frac{B}{k} \biggr) -\tau B. $$

If

$$ \tau \geq \frac{r ( b-k )^{2}}{4k}, $$

then $f ( B ) =0\Leftrightarrow B =0$; $\lim_{B \rightarrow \infty } f ( B ) =-\infty $. In this case, the bacteria will become extinct.

If

$$ \tau < \frac{r ( b - k )^{2}}{4 k}, $$

then we have two positive constants $a_{1} > b$ and $a_{2} > k$ given by

$$ a_{1} = \frac{r ( b + k ) - \sqrt{r^{2} ( b - k )^{2} -4 kr\tau }}{2 r}\quad \mbox{and}\quad a_{2} = \frac{r ( b + k ) + \sqrt{r^{2} ( b - k )^{2} -4 kr\tau }}{2 r} $$

such that:

- if $B ( t ) < a_{1}$, then the bacteria will become extinct;
- if $a_{1} < B ( t ) < a_{2}$, then the bacteria will exponentially increase up to $a_{2}$
- if $B ( t ) > a_{2}$, then the bacteria will exponentially decrease up to $a_{2}$,

where $a_{1}$ is the Allee threshold and $a_{2}$ is the carrying capacity. Hence Lemma 2.

Thus, for system (1)–(15), the population classes are positive and bounded. Hence, system (1)–(15) is well defined.

## Parameter estimation

Based on the real data collected from Yemen (January 1, 2020 to May 30, 2020), this work aims to study the outbreaks of COVID-19 and cholera in the same country. The COVID-19 data was obtained from the Center for Systems Science and Engineering (CSSE) at John Hopkins University (https://github.com/CSSEGISandData/COVID-19). The cholera data was obtained from the WHO and some data was taken from the health officials in Yemen. While the cholera data was taken weekly, that of COVID-19 was taken daily. Then the weekly data for both diseases was considered. Since the simultaneous data is required to compare and to determine the optimal policy, the data was standardized into weekly as that is more accurate than converting it into daily data.

To estimate the parameters, we used the least-squares regression approach, where we minimized the mean squared error (MSE) between prediction states and their available observation values derived from Yemen data according to Eq. (16):

16 $$ \min E_{0} = \sum_{l=0}^{N} w_{l} \bigl\Vert x_{l} - \hat{x_{l}} ( t ) \bigr\Vert ^{2}, $$

in which $w_{l}$ are normalization weights and $x_{l} $ and $\hat{x_{l}}$ represent the number of each epidemiological class and its predictions, respectively. Pyomo optimization modelling implemented in Python is employed to solve the least-squares regression problem. The orthogonal collocation method is used to discretize the dynamic differential equations system (1)–(15) taking into consideration the time domain which consists of weekly finite elements. The means of the estimated parameters are the following: $E_{0} =0.28499$, $\mu _{1} =0$, $\mu _{2} =0.00030$, $\mu _{12} =0$, $\beta =0.02325$, $\alpha _{c} ( t ) =0.25$, $\delta _{1} =0$, $\delta _{2} =0$, $\beta _{1} =0.005$, $\beta _{2} =1.5$, $\lambda _{1} =0$, $\lambda _{2} =0$, $\upsilon _{1} =0$, $\upsilon _{2} =0$, $\upsilon _{12} =0$, $\omega _{1} =0.04470$, $\omega _{12} =0$, $\omega _{2} =0.0002$, $\xi =40$, $\tau =0.333$, and $\alpha _{12} =0$. These estimated values of the parameters were obtained from historical data for both infections in Yemen (January 1, 2020 to May 30, 2020), and they will be used to solve the optimal control model in the next section. As the confirmed infected cases of co-infection simultaneously or consecutively in Yemen are still nonexistent, the parameter values of the dual infection equal zero. By comparing the estimated values of the parameters with the parameters estimated in [17], we find that the death rate due to COVID-19 in Yemen is so high at the rate of 0.0447, while the death rate due to COVID-19 in Germany is lower than other at the rate of 0.0024. According to [17] in some other countries, as the USA, the death rate is 0.0044, in Italy it is 0.010619, and in Spain it is 0.011871. Regarding the recovered cases, Germany occupies the first position at the rate of (0.046838), then comes Spain at the rate of 0.040129. In the third place comes Italy at the rate of 0.016644. The USA occupies the fourth position at the rate of 0.007467, and Yemen comes in the last position at the rate 0.005. According to [11] in 2017, while the death and recovery rates of cholera were 0.0003 and 1.4, respectively, in this study there is a slight improvement as the death and recovery rates of cholera are 0.0002 and 1.5, respectively.

**Figure 2:**
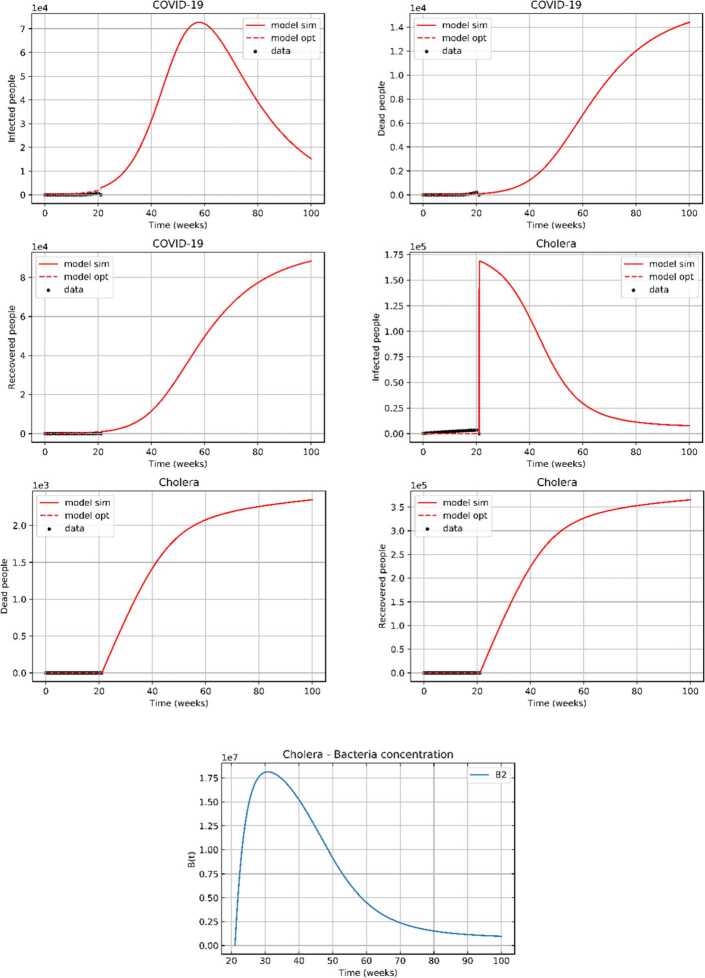
Analysis and week-wise prediction using the proposed model. The black dotted lines represent the real data, the red dotted lines represent the fitting line from the proposed model, and the red solid lines represent the predicted data (results) using the system

Furthermore, the predicted values of the infected, recovered, and deaths of both diseases were obtained, as illustrated in Fig. 2. Note that the mortality rate due to COVID-19 is very high compared to the mortality rate due to cholera even though the number of infected individuals with cholera is much higher than the number of infected individuals with COVID-19.

## Optimal control problem

Both epidemics are optimally controlled by minimizing the total number of infected individuals and this is done by controlling time-dynamic parameters appropriate for Yemen. The control policy that we propose aims at minimizing the number of exposed individuals to COVID-19 by controlling the parameters related to lockdown, social distancing, and the number of tests. The number of infected individuals with cholera can be minimized by increasing the distribution of CWTs for water purification for all susceptible individuals. This helps to minimize the number of infected individuals with COVID-19 or cholera or co-infections.

In our model, we consider four control functions $u_{1} ( t )$, $u_{2} ( t )$, $u_{3} ( t )$, and $u_{4} ( t ) $. The first $u_{1} ( t )$ represents the social distancing rate. This is an important parameter because it can be applied in Yemen by requiring people to distance socially, to wear masks, and to improve their personal hygiene. Therefore, $u_{1} ( t )$ assists us in examining its impact on reducing the COVID-19 outbreak. The second control function $u_{2} ( t )$ represents the quarantine and isolation rates. Most people in Yemen live on daily wages, which means that it is difficult to implement a complete lockdown on cities. Hence, only small values of this parameter are used. $u_{2} ( t )$ allows us to investigate its impact on curbing the COVID-19 outbreak. The third control function $u_{3} ( t )$ represents the number of COVID-19 test kits. By increasing the number of test kits, infected individuals can be discovered quickly, which means that they can be isolated and treated promptly. Therefore, $u_{3} ( t )$ allows us to investigate its impact on mitigating the spread of the COVID-19 epidemic. The last control function $u_{4} ( t )$ represents the fraction of susceptible individuals who can get CWTs for water purification. By increasing $u_{4} ( t )$, the water will be purer and the vector cholera and other diseases will be eliminated. Therefore, $u_{4} ( t )$ allows us to investigate its impact on curbing the cholera outbreak.

The objective function of the proposed model is to minimize the total number of new infected cases with both diseases as well as the cost associated with each control over a particular period of time. The objective function is formulated as follows:

17 $$\begin{aligned} & \min_{u_{i} ( t ),i=1,..,4} \int _{t_{0}}^{t_{f}} z_{1} I_{1} ( t ) + z_{2} I_{2} ( t ) + z_{3} I_{12} ( t ) \\ &\quad {} + D_{1} \bigl( u_{1} ( t ) \bigr)^{2} + D_{2} \bigl( u_{2} ( t ) \bigr)^{2}+ D_{3} \bigl( u_{3} ( t ) \bigr)^{2} + D_{4} \bigl( u_{4} ( t ) \bigr)^{2}, \end{aligned}$$

18 $$\begin{aligned} & \mbox{(1--15)}, \\ & \max_{t} \bigl( I_{l} ( t ) \bigr) \leq I_{l}^{\mathrm{peak}},\quad l=1,2, \end{aligned}$$

19 $$\begin{aligned} & u_{1} ( t ) \in [ 0.05,0.5 ], \end{aligned}$$

20 $$\begin{aligned} & u_{2} ( t ) \in [ 0.01,0.3 ], \end{aligned}$$

21 $$\begin{aligned} & u_{3} ( t ) \in [ 0.1,0.3 ], \end{aligned}$$

22 $$\begin{aligned} & u_{4} ( t ) \in [ 0,1 ] . \end{aligned}$$

The objective function in Eq. (17) minimizes the number of individuals infected with COVID-19, cholera, and both. It also minimizes the total costs associated with the control interventions. The constraints of the optimal control model are incorporated in Eqs. (1)–(15). The bound of the time-dependent control variables and the infected individuals for each infection must be under the estimated peaks for each infection. Constraint (18) determines the limit of the peak size for each epidemic. Constraints (19)–(22) determine the ranges of the time-dynamic parameters, where $z_{1}$, $z_{2}$, $z_{3}$, $D_{1}$, $D_{2}$, $D_{3}$, and $D_{4}$ are weight coefficients. The optimal control model is assumed during the full-time horizon $[ t_{0}, t_{f} ]$, where $t_{f}$ is 100 weeks.

Therefore, the solution of the optimal control model is $( u_{1}^{*} ( t ), u_{2}^{*} ( t ), u_{3}^{*} ( t ), u_{4}^{*} ( t ) )$ in which

23 $$ J \bigl( u_{1}^{*} ( t ), u_{2}^{*} ( t ), u_{3}^{*} ( t ), u_{4}^{*} ( t ) \bigr) = \min \bigl\{ J \bigl( u_{1} ( t ), u_{2} ( t ), u_{3} ( t ), u_{4} ( t ) \bigr) \vert u_{i} ( t ) \in \Psi , i =1,\dots 4 \bigr\} , $$

where $\Psi = ( u_{1} ( t ), u_{2} ( t ), u_{3} ( t ), u_{4} ( t ) )$ and $0\leq u_{1} ( t ) \leq 0.5$, $0\leq u_{2} ( t ) \leq 0.3$, $0\leq u_{3} ( t ) \leq 0.3$, $0\leq u_{4} ( t ) \leq 1$

Pontryagin’s maximum principle is an indirect method to solve the optimal control model through derivation of the Hamiltonian function and defining the necessary conditions for the optimal control of the cholera-COVID-19 co-infection model. The Hamiltonian *H* is defined as follows:

24 $$ \begin{aligned} H&= z_{1} I_{1} ( t ) + z_{2} I_{2} ( t ) + z_{3} I_{12} ( t ) + D_{1} \bigl( u_{1} ( t ) \bigr)^{2} + D_{2} \bigl( u_{2} ( t ) \bigr)^{2} + D_{3} \bigl( u_{3} ( t ) \bigr)^{2} \\ &\quad {}+ D_{4} \bigl( u_{4} ( t ) \bigr)^{2}+ M_{S} \biggl( \mu _{1} R_{1} ( t ) + \mu _{2} R_{2} ( t ) + \mu _{12} R_{12} ( t ) + \Lambda - \biggl( \frac{ ( 1- u_{1} ( t ) )}{N} A ( t )\\ &\quad {} + \frac{ ( 1- u_{2} ( t ) )}{N} I_{1} ( t ) + \alpha _{c} ( t ) I_{2} ( t ) + \frac{\beta B}{K_{B} +B} \bigl( 1- u_{4} ( t ) \bigr) + \alpha _{12} \biggr) S ( t ) \biggr) \\ &\quad {}+ M_{E} \biggl( \biggl( \frac{ ( 1- u_{1} ( t ) )}{N} A ( t ) + \frac{ ( 1- u_{2} ( t ) )}{N} I_{1} ( t ) \biggr) S ( t ) - t_{\mathrm{latent}}^{-1} E ( t ) \biggr)\\ &\quad {} + M_{A} \bigl( t_{\mathrm{latent}}^{-1} E ( t ) - \bigl( u_{3} ( t ) +\rho \bigr) A ( t ) \bigr) + M_{I_{1}} \bigl( u_{3} ( t ) A ( t ) - ( \delta _{1} + \beta _{1} + \omega _{1} ) I_{1} ( t ) \bigr)\\ &\quad {} + M_{R_{1}} \bigl( \rho A ( t ) + \beta _{1} I_{1} ( t ) - ( \mu _{1} + \lambda _{1} ) R_{1} ( t ) \bigr) + M_{P_{1}} \bigl( \omega _{1} I_{1} ( t ) \bigr) + M_{I_{R2}} \bigl( \lambda _{1} R_{1} ( t )\\ &\quad {} - \upsilon _{1} I_{R2} ( t ) \bigr) + M_{I_{12}} \bigl( \alpha _{12} S ( t ) + \delta _{1} I_{1} ( t ) + \delta _{2} I_{2} ( t ) - ( \upsilon _{12} + \omega _{12} ) I_{12} ( t ) \bigr)\\ &\quad {} + M_{R_{12}} \bigl( \upsilon _{1} I_{R2} ( t ) + \upsilon _{2} I_{1R} ( t ) + \upsilon _{12} I_{12} ( t ) - \mu _{12} R_{12} ( t ) \bigr) + M_{P_{12}} \bigl( \omega _{12} I_{12} ( t ) \bigr)\\ &\quad {} + M_{I_{2}} \biggl( \biggl( \alpha _{c} ( t ) I_{2} ( t ) + \frac{\beta B}{K_{B} +B} \bigl( 1- u_{4} ( t ) \bigr) \biggr) S ( t ) - ( \delta _{2} + \beta _{2} + \omega _{2} +\xi ) I_{2} ( t ) \biggr)\\ &\quad {} + M_{B} \biggl( rB ( B-b ) \biggl( 1- \frac{B}{k} \biggr) -\tau B+ \xi I_{2} ( t ) \biggr) + M_{R_{2}} \bigl( \beta _{2} I_{2} ( t ) - ( \mu _{2} + \lambda _{2} ) R_{2} ( t ) \bigr)\\ &\quad {} + M_{P_{2}} \bigl( \omega _{2} I_{2} ( t ) \bigr) + M_{I_{1R}} \bigl( \lambda _{2} R_{2} ( t ) - \upsilon _{2} I_{1R} ( t ) \bigr), \end{aligned} $$

where $M_{S}$, $M_{E}$, $M_{A}$, $M_{I_{1}}$, $M_{R_{1}}$, $M_{P_{1}}$, $M_{I_{R2}}$, $M_{I_{12}}$, $M_{R_{12}}$, $M_{P_{12}}$, $M_{I_{2}}$, $M_{B}$, $M_{R_{2}}$, $M_{P_{2}}$, and $M_{I_{1R}}$ are adjoint variables or co-state variables.

### Theorem

*Given the optimal control* $u_{1}^{*} ( t )$, $u_{2}^{*} ( t )$, $u_{3}^{*} ( t )$, $u_{4}^{*} ( t )$ *and solutions* *S*, *E*, *A*, $I_{1}$, $R_{1}$, $P_{1}$, $I_{R2}$, $I_{12}$, $R_{12}$, $P_{12}$, $I_{2}$, *B*, $R_{2}$, $P_{2}$, *and* $I_{1R}$ *of the corresponding state system* (1)*–*(15) *that minimize* $J ( u_{1} ( t ), u_{2} ( t ), u_{3} ( t ), u_{4} ( t ) )$ *over* Ψ. *There exist adjoint variables* $M_{S}$, $M_{E}$, $M_{A}$, $M_{I_{1}}$, $M_{R_{1}}$, $M_{P_{1}}$, $M_{I_{R2}}$, $M_{I_{12}}$, $M_{R_{12}}$, $M_{P_{12}}$, $M_{I_{2}}$, $M_{B}$, $M_{R_{2}}$, $M_{P_{2}}$, *and* $M_{I_{1R}}$ *such that*

25 $$ \frac{-d M_{l}}{dt} = \frac{\partial H}{\partial l}, $$

*where* $l=S, E, A, I_{1}, R_{1}, P_{1}, I_{R2}, I_{12}, R_{12}, P_{12}, I_{2}, B, R_{2}, P_{2},\textit{ and }I_{1R}$ *with the transversality conditions*

$$ \begin{aligned} M_{S} ( t_{f} ) &= M_{E} ( t_{f} ) = M_{A} ( t_{f} ) = M_{I_{1}} ( t_{f} ) = M_{R_{1}} ( t_{f} ) = M_{P_{1}} ( t_{f} ) = M_{I_{R2}} ( t_{f} ) = M_{I_{12}} ( t_{f} )\\ & = M_{R_{12}} ( t_{f} ) = M_{P_{12}} ( t_{f} ) = M_{I_{2}} ( t_{f} ) = M_{B} ( t_{f} ) = M_{R_{2}} ( t_{f} ) = M_{P_{2}} ( t_{f} ) = M_{I_{1R}} ( t_{f} ) =0 \end{aligned} $$

*and*

26 $$ \begin{gathered} u_{1}^{*} ( t ) = \min \biggl\{ 1, \max \biggl( 0, \frac{ ( M_{E} - M_{S} ) A ( t ) S ( t )}{2 D_{1} N} \biggr) \biggr\} ,\\ u_{2}^{*} ( t ) = \min \biggl\{ 1, \max \biggl( 0, \frac{ ( M_{E} - M_{S} ) I_{1} ( t ) S ( t )}{2 D_{2} N} \biggr) \biggr\} ,\\ u_{3}^{*} ( t ) = \min \biggl\{ 1, \max \biggl( 0, \frac{ ( M_{A} - M_{I_{1}} ) A ( t )}{2 D_{3}} \biggr) \biggr\} ,\\ u_{4}^{*} ( t ) = \min \biggl\{ 1, \max \biggl( 0, \frac{ ( M_{I_{2}} - M_{S} ) \beta BS ( t )}{2 D_{4} ( K_{B} +B )} \biggr) \biggr\} . \end{gathered} $$

The proof of this theorem is in Appendix A.

## Numerical simulations

In this section, we investigate and analyze the impact of the four dynamic control measures and the peak limits, which will determine the best policy for curbing the rapid spread of cholera and COVID-19 in Yemen in 2020. The policy approach is based on monotonous declines over time for quarantine, social separation, and monotonic increases for both the number of COVID-19 tests and the number of CWTs for water purification. Pyomo optimization modelling in Python was used to solve the optimal control model (16)–(21). The orthogonal collocation method is again used to discretize the dynamic differential equations system (1)–(15) taking into consideration the time domain which consists of weekly finite elements. Parameter values obtained from the solution of the parameters estimation problem in Sect. 3 are used for solving the optimal control model. Moreover, the other parameters and initial conditions in the optimal control model are fixed as indicated in Table 1 and Table 2 for all the following simulations. Table 3 illustrates the range of the time-dynamic functions for each policy. The following policies are discussed.

**Table 3:** Ranges of control parameters and values of limit peaks for each policy

Policy	$( [ \alpha _{a}^{l}, \alpha _{a}^{u} ], [ \alpha _{i}^{l}, \alpha _{i}^{u} ], [ \kappa ^{l}, \kappa ^{u} ], [ u^{l}, u^{u} ] )$	$( I_{1}^{\mathrm{peak}}, I_{2}^{\mathrm{peak}} )$	The associated cost
No control	([0,0],[0,0],[0,0],[0,0])	(10^5^,5 × 10^5^)	–
Med control	([0.2,0.4],[0.1,0.2],[0.2,0.25],[0.25,0.75])	(10^4^,5 × 10^5^)	4.5349228133
Max control	([0.4,0.5],[0.25,0.3],[0.25,0.3],[0.75,1])	(10^4^,5 × 10^5^)	2.8707575932
Mix control	([0.2,0.4],[0,0.1],[0.2,0.3],[0.7,0.9])	(10^4^,5 × 10^5^)	1.9432928849
Min peaks	([0.4,0.5],[0.1,0.2],[0.15,0.2],[025,0.95])	(10^4^,5 × 10^5^)	0.16289408246
Max peaks	([0.4,0.5],[0.1,0.2],[0.15,0.2],[0.25,0.95])	(10^5^,5 × 10^5^)	13.678641590

*Policy 1: (No control) Baseline*

In this policy, all coefficients of the time-dynamic variables in the objective function and the constraints are assumed to be equal to zero. In addition, the limit of the peaks for both outbreaks is equal to the maximum peaks indicated in Table 3.

The obtained results of the numerical simulations show that this combination is not good to curb both outbreaks. From the panels of the infected individuals with both epidemics in Fig. 4, Appendix B, we note that the peaks infected individuals with COVID-19 and cholera are high and their curves take too long to come down. Similarly, the panel of the bacteria population shows that free bacteria growth is the highest in growth and the slowest to decline over time.

*Policy 2: (Med control) Sufficient resources for both CWT distribution and test kits with average social distancing and quarantining*

In this policy, we assume that test kits are in the range of 20%–25% and are sufficient for the number of individuals susceptible to COVID-19. We also assume that CWTs are distributed to 25%–75% of the individuals susceptible to cholera. Moreover, the average values of the proportions of social distancing and quarantining are considered. Quarantining is applied only with a ratio of less than 20% and social distancing is applied with a ratio of less than 40%.

The results are shown in Fig. 5 in Appendix B. From the control time-dynamic functions panel, we observe that all time-dynamic functions have changed dynamically in the defined range referred to in Table 3. However, the results for this policy improve compared to those for the baseline policy, especially in terms of the number of infected individuals over time for both epidemics, the epidemic periods, and the bacteria growth.

*Policy 3: (Max control) Abundant resources for both CWT distribution and test kits with strict social distancing and quarantining*

In this strategy, we assume that almost all individuals susceptible to cholera have access to pure water and that test kits are available for almost all individuals susceptible to COVID-19. Moreover, strict rules for social distancing and quarantining are assumed in this policy.

The results are shown in Fig. 6 in Appendix B. From the control time-dynamic functions panel, we observe that while $u_{4} ( t )$ and $u_{3} ( t )$ increase monotonically, $u_{1} ( t )$ and $u_{2} ( t )$ decrease monotonically. Moreover, the fitted lines almost match the real data for both infections. This policy can affect the curve peaks for both epidemics and can end the epidemic period. Also, this policy can inhibit the rapid growth of bacteria.

*Policy 4: (Mix control) Abundant resources for CWT distribution and sufficient resources for test kits with average social distancing and low quarantining*

In this policy, we assume that almost all individuals susceptible to cholera have access to pure water and that test kits are available for a sufficient number of individuals susceptible to COVID-19. In addition, we assume that average social distancing is applied with less quarantining. From Fig. 7, Appendix B, we note that there are slight changes in the results of this policy compared to those of the Max control policy in terms of the number of cases for each epidemiological class, the peaks, and the speed of ending the epidemic. The results indicate the importance of increasing $u_{3} ( t )$ and $u_{4} ( t )$ and imposing social distancing $u_{1} ( t )$, and in return the lack of complete social quarantine $u_{2} ( t )$, since it has negative economic effects.

*Policy 5: Min peaks*

We assumed in all the aforementioned policies that the peak of COVID-19 does not exceed 100,000 and that the peak of cholera does not exceed 300,000. In this policy, we change the peak of COVID-19 to be less than 10,000 and the peak of cholera be less than 100,000. The ranges of the time-dynamic measures used in this policy are shown in Table 3.

We can see from the results in Fig. 8, Appendix B that the number of infected individuals for each epidemic is not high, while maintaining the same pattern of results with the previous policies.

*Policy 6: Max peaks*

In this scenario, we investigate the effect of limit values of peaks on the numbers of each epidemic class by increasing the limits of the peak values as indicated in Table 3.

In Fig. 9, Appendix B a significant increase in all the numbers of COVID-19 epidemic classes and a slight increase in the cholera epidemic classes are noticed.

We summarize the range of the control parameters for each policy, the values of the limit peaks, and the obtained cost of all simulations in Table 3. We can observe clearly that policy 5 (Min peaks) has minimum cost with an objective function value of 0.16289408246.

**Figure 3:**
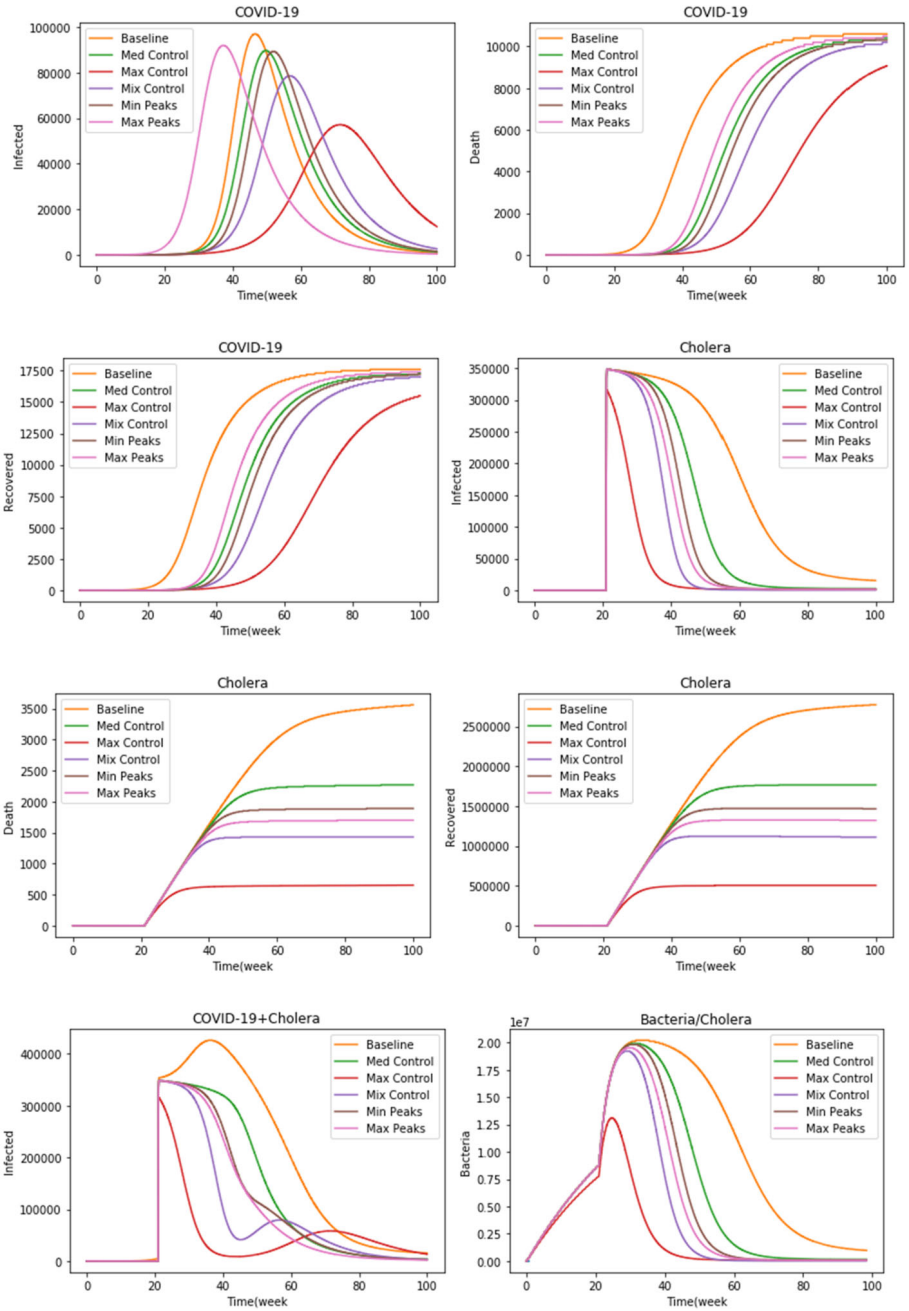
Comparison of the simulation results of the policies for all epidemiological classes

**Figure 4:**
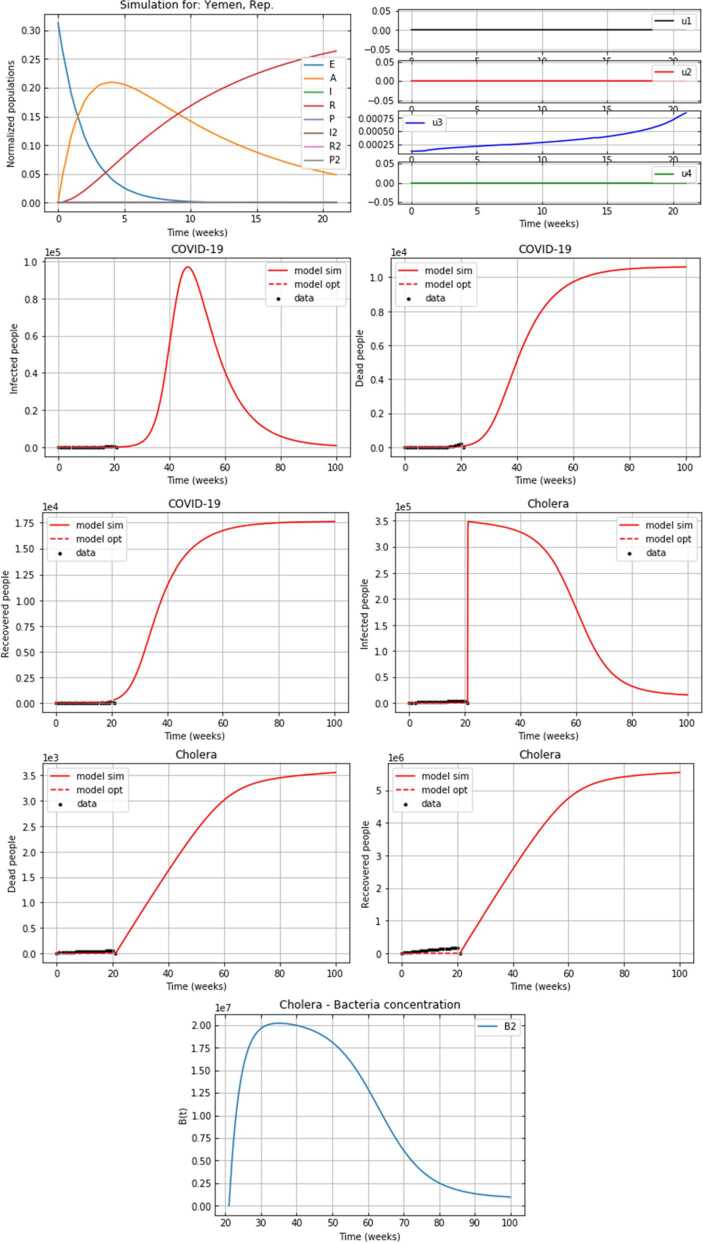
Results for policy 1

**Figure 5:**
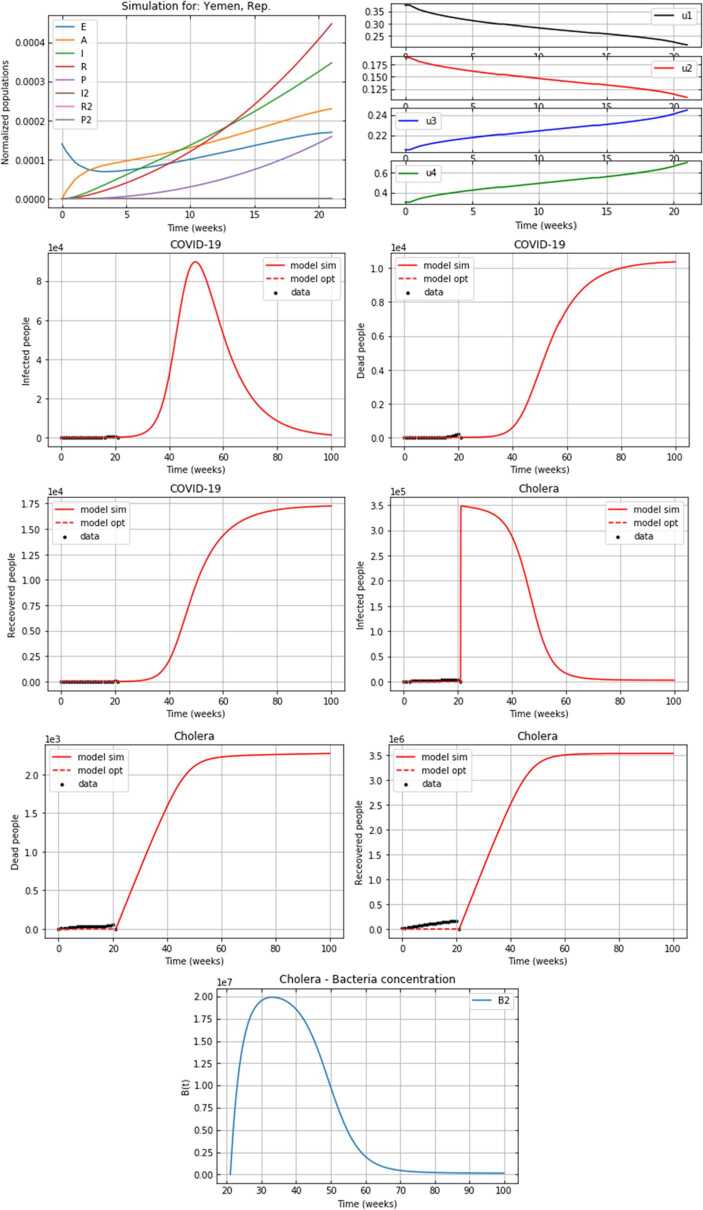
Results for policy 2

**Figure 6:**
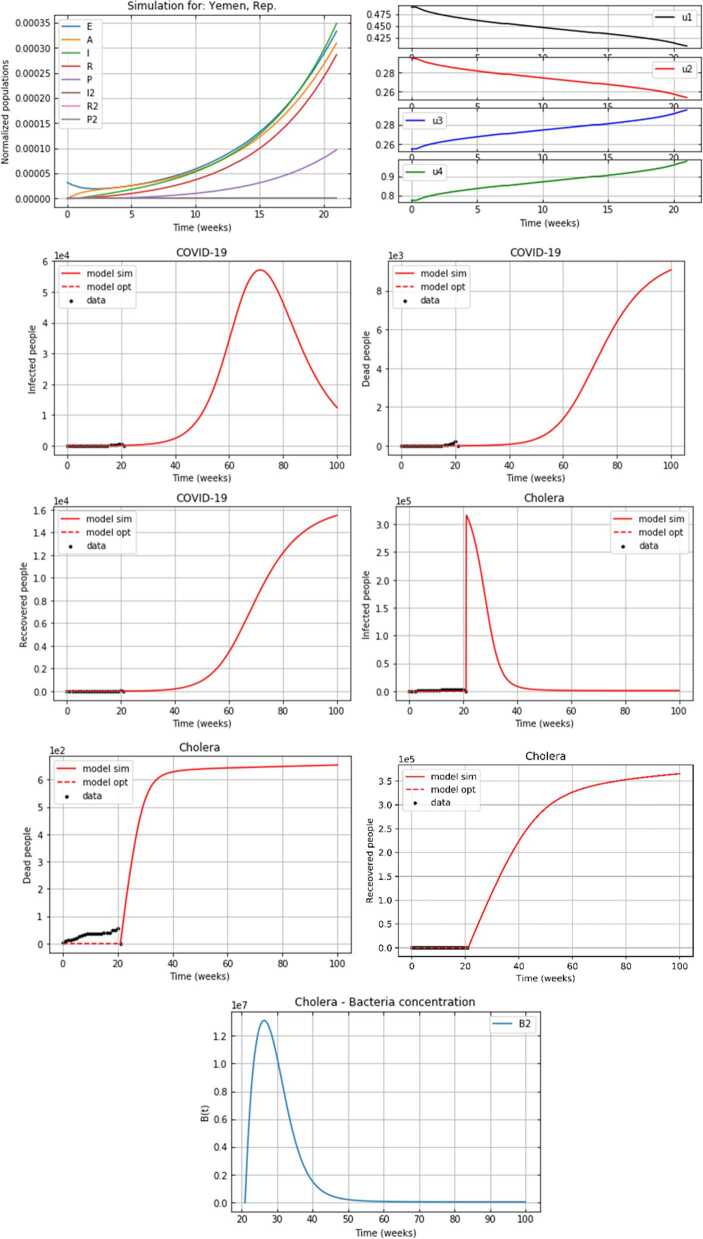
Results for policy 3

**Figure 7:**
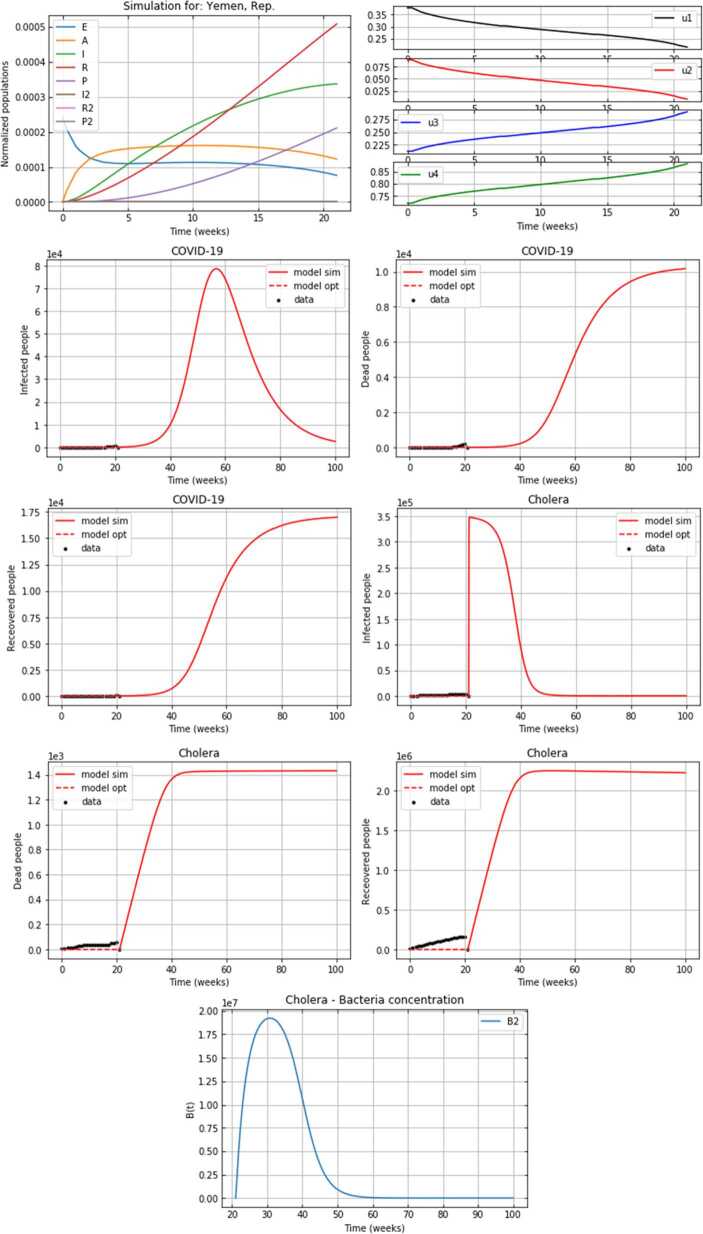
Results for policy 4

**Figure 8:**
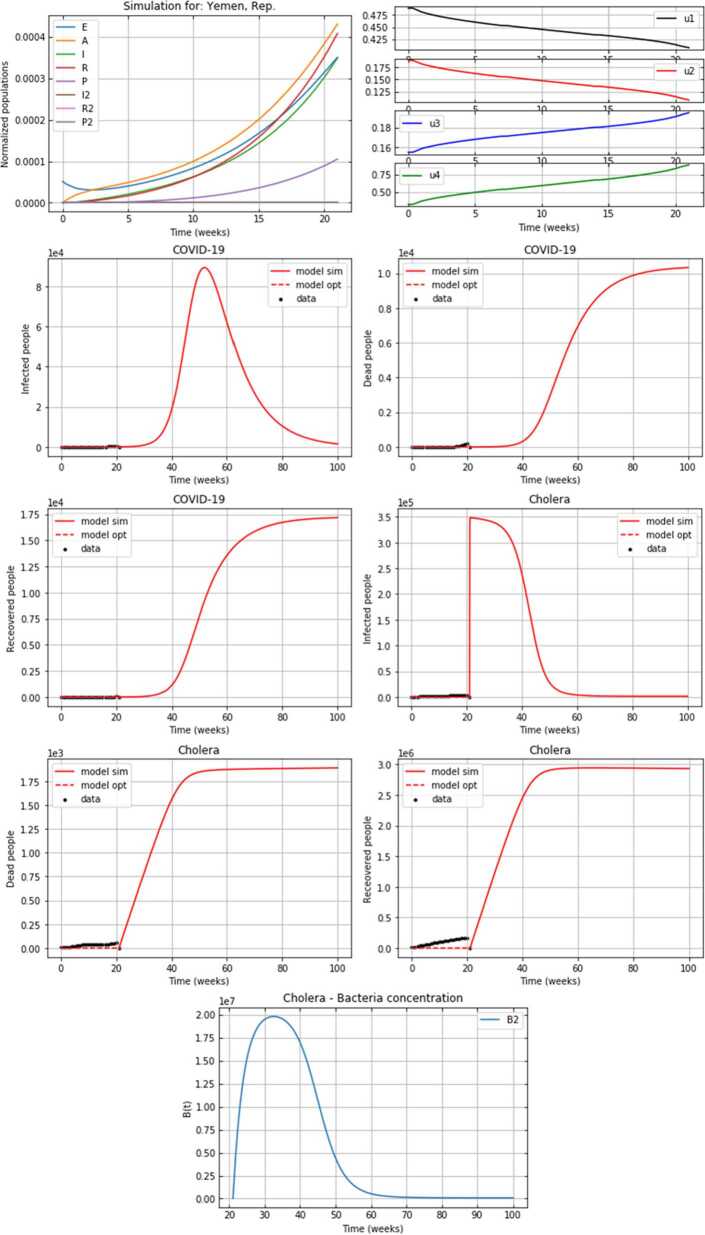
Results for policy 5

**Figure 9:**
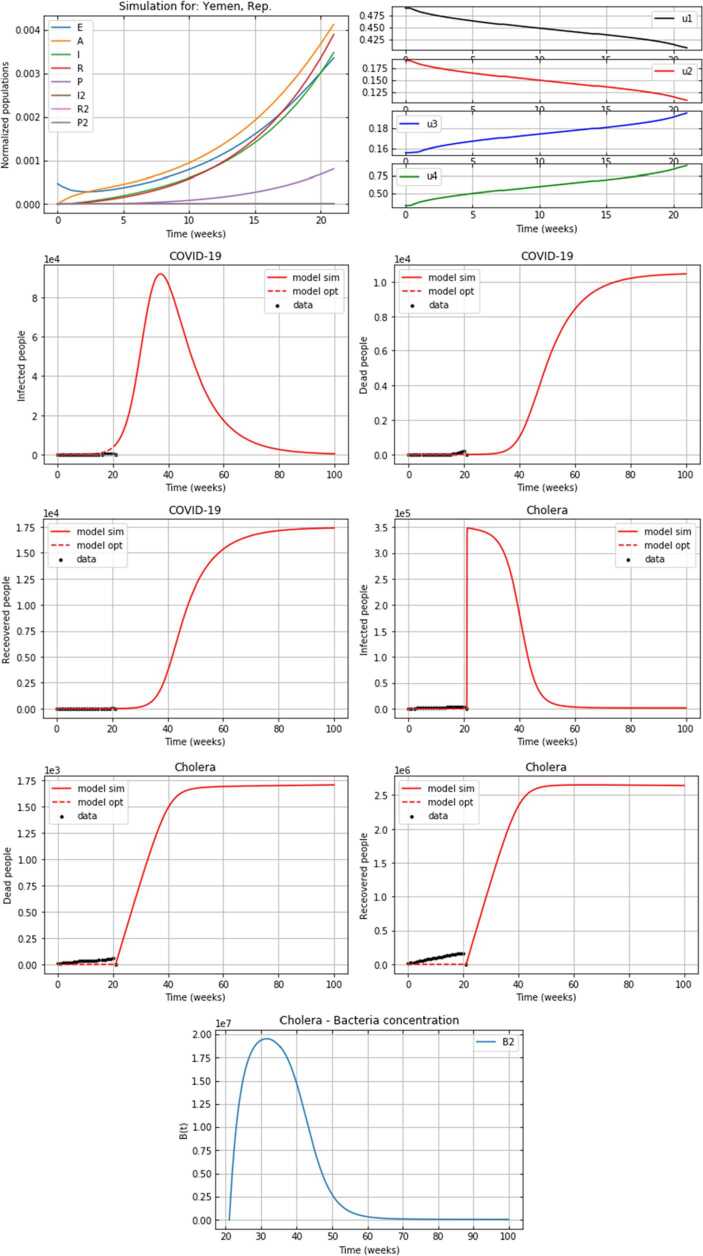
Results for policy 6

Figure 3 shows the comparison of the simulation results of the different policies for all the epidemiological classes. Having a glance at the panel of infected individuals with COVID-19, it is clear that policy 3 (Max control) leads to a flatter epidemic curve and the lowering in the number of infected individuals. In the second rank comes policy 4 (Mix control), which corresponds to distributing CWTs to all individuals susceptible to cholera, providing a sufficient number of test kits with a reasonable commitment to social distancing, and quarantining only for the infected individuals with COVID-19. Moreover, policy 1 (No control) leads to the highest peak size.

On the other side, having a look at the panel of perished individuals due to COVID-19, it is shown that policy 1 (No control) is the worst among all the other policies. Nevertheless, policy 3 with Max control leads to the lowest number of people lost due to COVID-19. The panel of recovered individuals from COVID-19 shows that policy 1 (No control) leads to the highest number of recovered individuals as compared to other policies. This was expected due to the increase in the number of infected individuals with COVID-19.

The panel of the infected individuals with cholera shows that policy 3 (Max control) is the best to reduce the number of infected individuals as well as the fastest in ending the epidemic. Policy 1 (Baseline) is the worst in terms of the number of infected individuals having the highest peak size. In addition, it has also the longest epidemic period. The panel of the perished individuals due to cholera shows that while the first and second policies (Baseline and Med control) lead to the highest numbers, the third and fourth policies (Max and Mix control) lead to the lowest numbers. Conversely, the panel of the recovered individuals from cholera shows that while the first and second policies (Baseline and Med control) lead to the highest number of the recovered individuals, the third and fourth policies (Max and Mix control) lead to the lowest number of the recovered individuals due to the large numbers of infected individuals with cholera.

The panel of the bacteria population shows that policy 3 (Max control) has the lowest peak and leads to the rapid decrease of the bacterial population growth. This contributes to reducing the number of individuals susceptible to cholera. Policy 4 (Mix control) comes in the second rank in terms of the peak and the return of the bacterial population curve. Our results show that while some policies are efficient in reducing the number of individuals infected with COVID-19, others are efficient in controlling the number of individuals infected with cholera (the ranks of the policies according to epidemiological class are shown in Table 4, Appendix C).

To determine the optimal policy that can be applied in Yemen, we can combine the co-infected cases of each policy. The results are shown in the panel of the infected individuals with COVID-19 + cholera. The third policy (Max control) is shown as the best among all the policies proposed to control and reduce the number of infected people with both epidemics. As applying a complete quarantine in a poor country like Yemen is not possible for its people earn their living on daily wage and lack awareness of the dangers of these epidemics, it is concluded that the most effective policy that can be carried out in Yemen is policy 4 (Mix control) since it seeks to increase the number of CWTs for water purification to almost all the individuals susceptible to cholera and to increase the number of test kits for the exposed individuals to COVID-19. Besides, reasonable social distancing has to be applied with quarantining for only infected cases.

## Conclusions

In the present work, we formulated a compartmental cholera-COVID-19 co-infection model that describes the dynamics of transmission of COVID-19 and cholera in Yemen. Then, we estimated the parameters of the model and proposed an optimal control policy to minimize the number of individuals infected with both infections and to minimize the total cost associated with each control. Then, optimal control policies were investigated. Those include four control functions: social distancing, lockdown, the number of test kits to control the COVID-19 outbreak, and the number of susceptible individuals who can get CWTs for water purification. We investigated the advantages of each policy by comparing the results of the numerical simulations. We demonstrated that the optimal policy to be applied in Yemen is based on social distancing to protect susceptible individuals from the infections, increasing the number of test kits for infected individuals, and increasing the number of CWTs for water purification. For future work, dual COVID-19 with other infections can be considered and other time-dynamic functions for controlling the infection outbreaks can be added.

## Data Availability

Not applicable.

## Appendix A

In this appendix, we prove the theorem in Sect. 4.

### Proof

By applying Pontryagin’s maximum principle to the Hamiltonian *H*, we get the following adjoint systems:

27 $$\begin{aligned}& \begin{aligned} \frac{d M_{S}}{dt} &=- \frac{\partial H}{\partial S} \\ &= ( M_{S} - M_{E} ) \biggl( \frac{ ( 1- u_{1} ( t ) )}{N} A ( t ) + \frac{ ( 1- u_{2} ( t ) )}{N} I_{1} ( t ) \biggr)\\ &\quad {} + ( M_{S} - M_{I_{2}} ) \biggl( \alpha _{c} ( t ) I_{2} ( t ) + \frac{\beta B}{K_{B} +B} \bigl( 1- u_{4} ( t ) \bigr) \biggr) + \alpha _{12} ( M_{S} - M_{I_{12}} ), \end{aligned} \\& \frac{d M_{E}}{dt} =- \frac{\partial H}{\partial E} = ( M_{E} - M_{A} ) t_{\mathrm{latent}}^{-1}, \\& \frac{d M_{A}}{dt} =- \frac{\partial H}{\partial A} = ( M_{S} - M_{E} ) \biggl( \frac{ ( 1- u_{1} ( t ) )}{N} \biggr) S ( t ) + u_{3} ( t ) ( M_{A} - M_{I_{1}} ) +\rho ( M_{A} - M_{R_{1}} ), \\& \begin{aligned} \frac{d M_{I_{1}}}{dt} &=- \frac{\partial H}{\partial I_{1}} \\ &=- z_{1} + ( M_{S} - M_{E} ) \biggl( \frac{u_{2} ( t ) S ( t )}{N} \biggr) \\ &\quad {}+ \beta _{1} ( M_{I_{1}} - M_{R_{1}} ) + \omega _{1} ( M_{I_{1}} - M_{P_{1}} ) + \delta _{1} ( M_{I_{1}} - M_{I_{12}} ), \end{aligned} \\& \frac{d M_{R_{1}}}{dt} =- \frac{\partial H}{\partial R_{1}} = ( M_{R_{1}} - M_{S} ) \mu _{1} + ( M_{R_{1}} - M_{I_{R2}} ) \lambda _{1}, \\& \frac{d M_{P_{1}}}{dt} =- \frac{\partial H}{\partial P_{1}} = 0, \\& \frac{d M_{I_{R2}}}{dt} =- \frac{\partial H}{\partial I_{R2}} = ( M_{I_{R2}} - M_{R_{12}} ) \nu _{1}, \\& \frac{d M_{I_{12}}}{dt} =- \frac{\partial H}{\partial I_{12}} =- z_{3} + ( M_{I_{12}} - M_{R_{12}} ) \nu _{12} + ( M_{I_{12}} - M_{P_{12}} ) \omega _{12}, \\& \frac{d M_{R_{12}}}{dt} =- \frac{\partial H}{\partial R_{12}} = ( M_{R_{12}} - M_{S} ) \mu _{12} , \\& \frac{d M_{P_{12}}}{dt} = \frac{\partial H}{\partial P_{12}} = 0, \\& \begin{aligned} \frac{d M_{I_{2}}}{dt} &=- \frac{\partial H}{\partial I_{2}} \\ &=- z_{2} + \alpha _{c} ( t ) S ( t ) ( M_{S} - M_{I_{2}} ) + \beta _{2} ( M_{I_{2}} - M_{R_{2}} ) + \omega _{2} ( M_{I_{2}} - M_{P_{2}} )\\ &\quad {} + \delta _{2} ( M_{I_{2}} - M_{I_{12}} ) +\xi ( M_{I_{2}} - M_{B} ), \end{aligned} \\& \frac{d M_{B}}{dt} =- \frac{\partial H}{\partial B} = M_{B} \tau + M_{B} \biggl( \frac{2Bb}{K_{B}} -3r B^{2} \biggr) + ( M_{B} - M_{I_{2}} ) \biggl( \frac{K_{B}}{ ( K_{B} +B )^{2}} \biggr) \bigl( 1- u_{4} ( t ) \bigr) S ( t ), \\& \frac{d M_{R_{2}}}{dt} =- \frac{\partial H}{\partial R_{2}} = ( M_{R_{2}} - M_{S} ) \mu _{2} + ( M_{R_{1}} - M_{I_{1R}} ) \lambda _{2}, \\& \frac{d M_{P_{2}}}{dt} = \frac{\partial H}{\partial P_{2}} = 0, \\& \frac{d M_{I_{1R}}}{dt} =- \frac{\partial H}{\partial I_{1R}} = ( M_{I_{1R}} - M_{R_{12}} ) \nu _{2}. \end{aligned}$$

We obtain the time-dependent function controls $u_{1}^{*} ( t )$, $u_{2}^{*} ( t )$, $u_{3}^{*} ( t )$, $u_{4}^{*} ( t )$ when $\frac{\partial H}{\partial u_{1} ( t )} = \frac{\partial H}{\partial u_{2} ( t )} = \frac{\partial H}{\partial u_{3} ( t )} = \frac{\partial H}{\partial u_{4} ( t )} =0$. Then, we obtain the following:

28 $$ \begin{gathered} \frac{\partial H}{\partial u_{1} ( t )} = 2 D_{1} u_{1} ( t ) + \frac{A ( t ) S ( t )}{N} ( M_{S} - M_{E} ) =0,\\ \frac{\partial H}{\partial u_{2} ( t )} = 2 D_{2} u_{2} ( t ) + M_{S} \frac{I_{1} ( t ) S ( t )}{N} - M_{E} \frac{I_{1} ( t ) S ( t )}{N} =0,\\ \frac{\partial H}{\partial u_{3} ( t )} = 2 D_{3} u_{3} ( t ) - M_{A} A ( t ) + M_{I_{1}} A ( t ) =0,\\ \frac{\partial H}{\partial u_{4} ( t )} = 2 D_{4} u_{4} ( t ) + M_{S} \frac{\beta BS ( t )}{K_{B} +B} - M_{I_{2}} \frac{\beta BS ( t )}{K_{B} +B} =0. \end{gathered} $$

Hence, we obtain

29 $$ \begin{gathered} u_{1}^{*} ( t ) = \frac{ ( M_{E} - M_{S} ) A ( t ) S ( t )}{2 D_{1} N},\\ u_{2}^{*} ( t ) = \frac{ ( M_{E} - M_{S} ) I_{1} ( t ) S ( t )}{2 D_{2} N},\\ u_{3}^{*} ( t ) = \frac{ ( M_{A} - M_{I_{1}} ) A ( t )}{2 D_{3}},\\ u_{4}^{*} ( t ) = \frac{ ( M_{I_{2}} - M_{S} ) \beta BS ( t )}{2 D_{4} ( K_{B} +B )}. \end{gathered} $$

By the standard control arguments involving the bounds on the controls, we conclude

30 $$ \begin{gathered} u_{1}^{*} ( t ) = \textstyle\begin{cases} 0 &\text{if } \frac{ ( M_{E} - M_{S} ) A ( t ) S ( t )}{2 D_{1} N} \leq 0, \\ \frac{ ( M_{E} - M_{S} ) A ( t ) S ( t )}{2 D_{1} N} &\text{if } 0< \frac{ ( M_{E} - M_{S} ) A ( t ) S ( t )}{2 D_{1} N} < 1,\\ 1 &\text{if } \frac{ ( M_{E} - M_{S} ) A ( t ) S ( t )}{2 D_{1} N} \geq 1, \end{cases}\displaystyle \\ u_{2}^{*} ( t ) = \textstyle\begin{cases} 0 &\text{if } \frac{ ( M_{E} - M_{S} ) I_{1} ( t ) S ( t )}{2 D_{2} N} \leq 0, \\ \frac{ ( M_{E} - M_{S} ) I_{1} ( t ) S ( t )}{2 D_{2} N} &\text{if } 0< \frac{ ( M_{E} - M_{S} ) I_{1} ( t ) S ( t )}{2 D_{2} N} < 1,\\ 1 &\text{if } \frac{ ( M_{E} - M_{S} ) I_{1} ( t ) S ( t )}{2 D_{2} N} \geq 1, \end{cases}\displaystyle \\ u_{3}^{*} ( t ) = \textstyle\begin{cases} 0 &\text{if } \frac{ ( M_{A} - M_{I_{1}} ) A ( t )}{2 D_{3}} \leq 0, \\ \frac{ ( M_{A} - M_{I_{1}} ) A ( t )}{2 D_{3}} &\text{if } 0< \frac{ ( M_{A} - M_{I_{1}} ) A ( t )}{2 D_{3}} < 1,\\ 1 &\text{if } \frac{ ( M_{A} - M_{I_{1}} ) A ( t )}{2 D_{3}} \geq 1, \end{cases}\displaystyle \\ u_{4}^{*} ( t ) = \textstyle\begin{cases} 0 &\text{if } \frac{ ( M_{I_{2}} - M_{S} ) \beta BS ( t )}{2 D_{4} ( K_{B} +B )} \leq 0, \\ \frac{ ( M_{I_{2}} - M_{S} ) \beta BS ( t )}{2 D_{4} ( K_{B} +B )} &\text{if } 0< \frac{ ( M_{I_{2}} - M_{S} ) \beta BS ( t )}{2 D_{4} ( K_{B} +B )} < 1,\\ 1 &\text{if } \frac{ ( M_{I_{2}} - M_{S} ) \beta BS ( t )}{2 D_{4} ( K_{B} +B )} \geq 1. \end{cases}\displaystyle \end{gathered} $$

 □

## Appendix B

In this appendix, we show the graphical results of all of the policies investigated in the study.

*Policy 1: (No control) Baseline*

*Policy 2 (Med control)*

*Policy 3: (Max control)*

*Policy 4: (Mix control)*

*Policy 5: Min peaks*

*Policy 6: Max peaks*

## Appendix C

**Table 4:** Ranks of the policies in various epidemiological classes

	$I_{1}$	$P_{1}$	$R_{1}$	$I_{2}$	$P_{2}$	$R_{2}$	$B_{2}$	$I_{1} + I_{2}$
1	Max control	Max control	Baseline	Max control	Max control	Baseline	Max control	Max control
2	Mix control	Mix control	Max peaks	Mix control	Mix control	Med control	Mix control	Mix control
3	Med control	Min peaks	Med control	Max peaks	Max peaks	Min peaks	Max peaks	Max peaks
4	Min peaks	Med control	Min peaks	Min peaks	Min peaks	Max peaks	Min peaks	Min peaks
5	Max peaks	Max peaks	Mix control	Med control	Med control	Mix control	Med control	Med control
6	Baseline	Baseline	Max control	Baseline	Baseline	Max control	Baseline	Baseline
